# Effective detection of biocatalysts with specified activity by using a hydrogel-based colourimetric assay – β-galactosidase case study

**DOI:** 10.1371/journal.pone.0205532

**Published:** 2018-10-11

**Authors:** Karolina Labus

**Affiliations:** Division of Bioprocess and Biomedical Engineering, Faculty of Chemistry, Wrocław University of Science and Technology, Wrocław, Poland; Brandeis University, UNITED STATES

## Abstract

The main aim of this study was to prepare gelatine-based hydrogels containing entrapped substrate and to examine the applicability of these matrices for detection of enzymes with a specified catalytic activity. The general research concept assumed the use of a substrate that, in the presence of a particular enzyme, will quickly undergo conversion to a coloured product. ortho-Nitrophenyl-β-D-galactopyranoside (ONPG) was used as the immobilized substrate and β-galactosidase from *Kluyveromyces lactis* as the biocatalyst to be determined. Among other factors, the range of detectable concentrations of galactosidase, the operational pH range, the time necessary to achieve a visible response and the preferred storage conditions for the test were determined. As a result, an effective colourimetric test for β-galactosidase detection was obtained. Its main advantages include (i) the effective detection of the enzyme at concentrations greater than or equal to 0.6 mg^.^L^-1^, (ii) the ability to perform initial quantification of the enzyme on the basis of the intensity of the obtained colour (iii) applicability in a wide pH range (from 4.0 to 9.0), (iv) a relatively short response time (from 1 to a maximum of 30 minutes) and (v) stability in long-term storage at 4°C (90 days without loss of specific properties).

## Introduction

The detection of biocatalysts of interest in multi-component mixtures, such as plant extracts or microbial post-culture fluids, is a notable challenge for modern biotechnology. The essential key to facilitating this task is to find a rapid, easy, and highly selective method for determining the catalytic activity. One possibility is the use of a substrate specific to one particular biocatalyst with a given activity. The most favourable would be an assay in which the product of the catalysed reaction provides an intense colour in a relatively short time.

Hydrogels are hydrophilic materials based on one or more types of polymer chains that form (as a result of crosslinking) a three-dimensional porous structure containing water in the voids between the individual building blocks. A diverse range of hydrophilic polymers and their combinations can be used for the production of hydrogels [1–4]. Thus, materials with the desired physical, chemical and biological features, such as an appropriate degree of swelling, packing density of the spatial structure, tensile properties, biocompatibility or biodegradation capacity, can be obtained. One of the most specific features of hydrogels that distinguishes them from other polymeric materials is the ability to swell in an aqueous microenvironment from 10- up to 1000-fold of their dried mass [5]. Other properties that make hydrogel matrices an object of interest in various commercial applications include (i) semi-permeability, (ii) the ability to create multilayer systems, which effectively mimic the organization of cells and tissues, (iii) the controllability of the crosslinking process (iv) biocompatibility and (v) bioresorption and biodegradation capability [5–7]. Due to these unique properties, these materials are particularly widespread in the biomedical sector in tissue scaffolds, wound dressings, implants, stents and surgical sutures [6–10]. A dynamic growth of interest in the application of hydrogel structures as supports for various bioactive molecules (e.g., drugs, growth factors) has also developed [9,11–14]. Currently, in this field, one of the intensively considered research issues is the examination of hydrogel matrices for enzyme immobilization [15,16]. Biocatalysts are immobilized using these materials most frequently by the entrapment method. Examples include: catalase [17], glucose oxidase [18], β-galactosidase [19], invertase [20], lipase [21], urease [22], tyrosinase [23] and laccase [24]. After appropriate covalent functionalization with chromo- or fluorogenic ligands, hydrogels can also be successfully used for the selective identification of pathogenic bacteria [25–27]. This solution is applicable in medicine in the construction of so-called ‘diagnostic dressings’ designed to identify bacterial infections that occur in the wound site. In this case the mechanism is based on monitoring virulence factors, specifically the enzymes characteristic of given bacterial strains [26,28]. To the best of the author's knowledge, the use of hydrogel matrices with immobilized substrates for the detection of enzymes with a specified catalytic activity in aqueous solution is a novel scientific issue that has not yet been reported in the available literature.

The main aim of this study was to prepare gelatine-based hydrogels containing entrapped substrate and to examine the applicability of these matrices in tests to detect enzymes with specified catalytic activity. The general research concept assumed the use of a substrate that, in the presence of a particular enzyme, will quickly undergo conversion to coloured products. The undoubted advantage of the proposed solution is the low cost of immobilization of the compounds in hydrogel structures by the entrapment method and the simplicity of performing the enzyme detection tests. Furthermore, the use of a substrate that is highly specific for a given enzyme enables the efficient detection of the desired biocatalyst, e.g., in post-culture microbial fluids, plant extracts or physiological fluids, which usually contain a complex mixture of proteins with different catalytic properties.

In this case study, ortho-nitrophenyl-β-D-galactopyranoside (ONPG) was used as the immobilized substrate and β-galactosidase from *Kluyveromyces lactis* as the biocatalyst to be determined. The enzyme selected for this research is a biocatalyst responsible for the hydrolysis of lactose that enables the consumption of milk and other dairy products by people with lactose intolerance [29–31]. Because this ailment affects the majority of the adult worldwide population, the development of a simple, rapid and highly selective assay for β-galactosidase determination could be of substantial interest in the medical diagnostics field. The proposed test for enzyme detection is so innovative that it has been successfully submitted for patent protection in Poland (application no. P421412).

## Materials and methods

### Materials

Porcine skin gelatine, *ortho*-nitrophenyl-β-D-galactopyranoside (ONPG) and β-galactosidase from *Kluyveromyces lactis* (EC 3.2.1.23) were purchased from Sigma-Aldrich (Germany). Microbial transglutaminase Activa®WM (mTGase) was kindly donated by Ajinomoto (Japan). Other reagents, all of analytical grade, were supplied by Avantor Performance Materials (Poland).

### Preparation of gelatine-based hydrogel matrices containing o-nitrophenyl-β-D-galactopyranoside (ONPG)

A weighed portion of gelatine (final concentration of 15% w/v) was dissolved in 0.1 M phosphate buffer at pH 7.5 in a thermostated reactor at 60°C for approximately 30 min. Next, the solution was cooled to 40°C and incubated at this temperature for a few minutes. In parallel, a buffer solution of mTGase (final concentration of 3.0% w/v) was prepared. The crosslinking was begun by mixing an appropriate amount of mTGase with gelatine solution at a volume ratio of 1:2 and afterward obtained blend was immediately cooled to 4°C and kept under these conditions for 24 hours. To achieve immobilization, a suitable amount of ONPG (final concentration 15 mM) was added to the gelatine prior to crosslinking step. As a result of crosslinking, the total amount of ONPG was immobilized in the hydrogel lattice. The final concentration of ONPG was selected based on previous kinetics experiments, where it has been shown that the ONPG concentration of 15 mM is about 17.4 Km and in this conditions the rate of β-galactosidase-catalyzed reaction is not dependent on the substrate concentration (S1 Appendix). Applied immobilization procedure was a slight modification of the method described previously [19].

### Hydrogel-based assay for the colourimetric determination of β-galactosidase

The test consists of applying 50 μL of a buffer solution potentially including β-galactosidase to the surface of gelatine hydrogel (200 μL) containing ortho-nitrophenyl-β-D-galactopyranoside (ONPG) placed in an Eppendorf tube. One then monitors the change in gel colour from transparent to yellow or the lack thereof. The yellow colour indicates the presence of β-galactosidase in the test solution, and the intensity of the yellow colour visually determined after a specified test time enables the preliminary estimation of the β-galactosidase amount.

### Determination of the β-galactosidase concentration range effectively detected by the hydrogel-based assay

First, β-galactosidase solutions of various concentrations (0.6–323 mg^.^L^-1^) were prepared in 0.1 M phosphate buffer with pH of 7.5. The protein concentrations in the enzyme solutions were determined by using the Lowry method [32]. Then, a small amount of each β-galactosidase preparation was applied to the gelatine-based hydrogel matrix containing ONPG, and the progress of the change in gel colour from transparent to yellow was monitored. In the research, it was assumed that a hydrogel matrix containing entrapped ONPG is effective for β-galactosidase detection and permits determination of the enzyme in a time not exceeding 30 min.

### Determination of the pH range in which β-galactosidase is effectively detected by using the hydrogel-based assay

First, β-galactosidase solutions at different pH (range of 4.0–9.0) were prepared in 0.1 M phosphate buffer. Then, a small amount of each β-galactosidase solution was applied to the gelatine-based hydrogel matrix containing ONPG, and with the passage of time, a change in gel colour from transparent to yellow was monitored. In the research, it was assumed that a test relying on a hydrogel containing entrapped ONPG is effective for β-galactosidase detection and permits determination of the enzyme in a time not exceeding 30 min.

### Storage stability of *ortho*-nitrophenyl-β-D-galactopyranoside

The stability of ONPG in native and immobilized form was verified after storage of the samples on the well plate at 4°C for specified period of time (1–20 days). The result for each ONPG formulation (solution or entrapped in hydrogel) stored for a particular time was compared with the results determined on day 0 (control sample)

### Storage stability of hydrogel-based test

The stability of gelatine hydrogel matrices containing ONPG was verified by performing a detection test for given concentration of β-galactosidase by using matrices that were previously stored at 4°C for a specified period of time (1–90 days) in sealed Eppendorf tubes. The result for each hydrogel matrix stored for a particular time was compared with the results determined on day 0 (control sample). For this purpose, the intensity of the colour change after 5 and 10 minutes was compared with that at the start of the galactosidase detection test. The hydrogel assay was classified as stable under storage conditions if the effect of the change in gel colour from transparent to yellow determined 10 min after the application of the enzyme solution at the same concentration (82 mg^.^L^-1^) was similar to that obtained for the control test performed on day 0.

## Results and discussion

**Fig 1:**
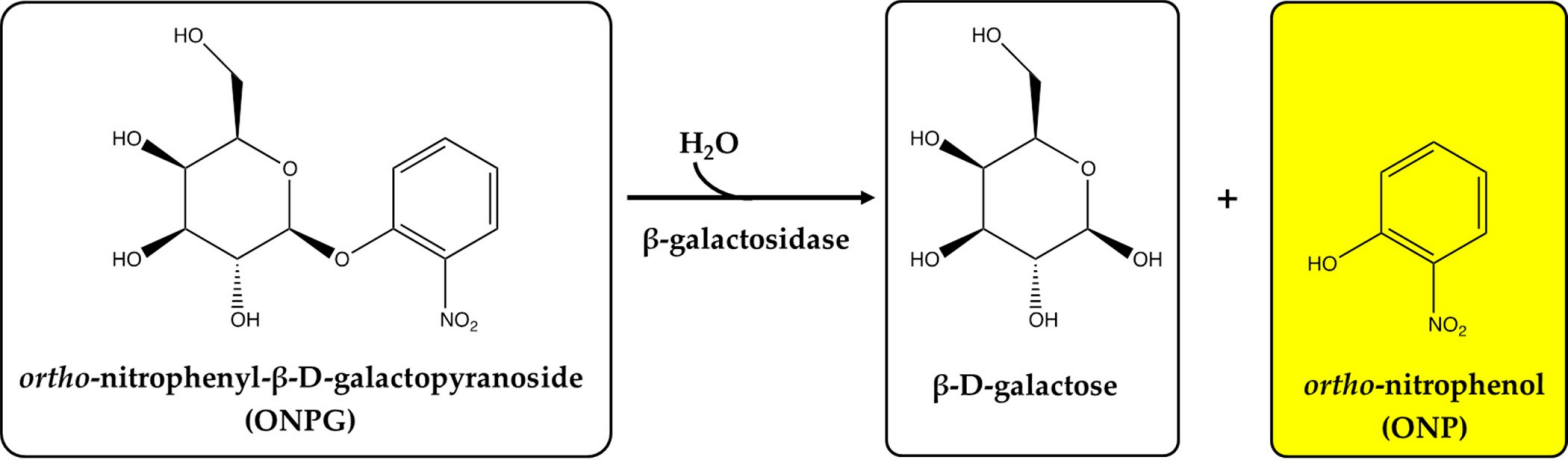
Reaction scheme of ortho-nitrophenyl-β-D-galactopyranoside (ONPG) hydrolysis in the presence of β-galactosidase as a biocatalyst.

The colourimetric test for β-galactosidase detection proposed in this study is based on the use of a synthetic substrate specific for this enzyme, ONPG, immobilized by entrapment inside the crosslinked structure of a hydrogel matrix. This compound is well known and is commonly used to determine the catalytic activity of β-galactosidase [33–35]. The presence of this biocatalyst is indicated by the appearance of a yellow colour as a result of the enzymatic hydrolysis of ONPG. In this case, o-nitrophenol (a product of ONPG decomposition catalysed by β-galactosidase) is responsible for the colour (Fig 1).

**Fig 2:**
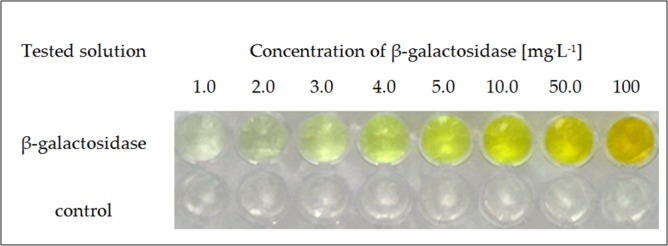
Response obtained in tests of β-galactosidase solutions at different concentrations; test time 10 min; enzyme concentrations 1.0–100 mg^.^L^-1^.

Briefly, a colourimetric test for the detection of the given enzyme was performed as follows: a small amount of solution potentially containing β-galactosidase was applied to the surface of a transparent hydrogel matrix containing colourless ONPG, and over time the colour change from transparent to yellow was monitored. The appearance of the colour indicates the presence of β-galactosidase in the tested solution. The intensity of the colour observed visually after a certain period of time allows a general estimation of the concentration of the detected enzyme. Examples of the results of the test performed at different concentrations of β-galactosidase are shown in Fig 2.

The ONPG-containing matrices may be prepared in various forms. For example in the form of a thin layer/drop on the glass plate, or as a certain volume of gel in an Eppendorf tube or a well plate. Nevertheless, the most preferably is to use the test prepared in an Eppendorf tube because there is no air access during storage and ONPG does not oxidize itself.

**Fig 3:**
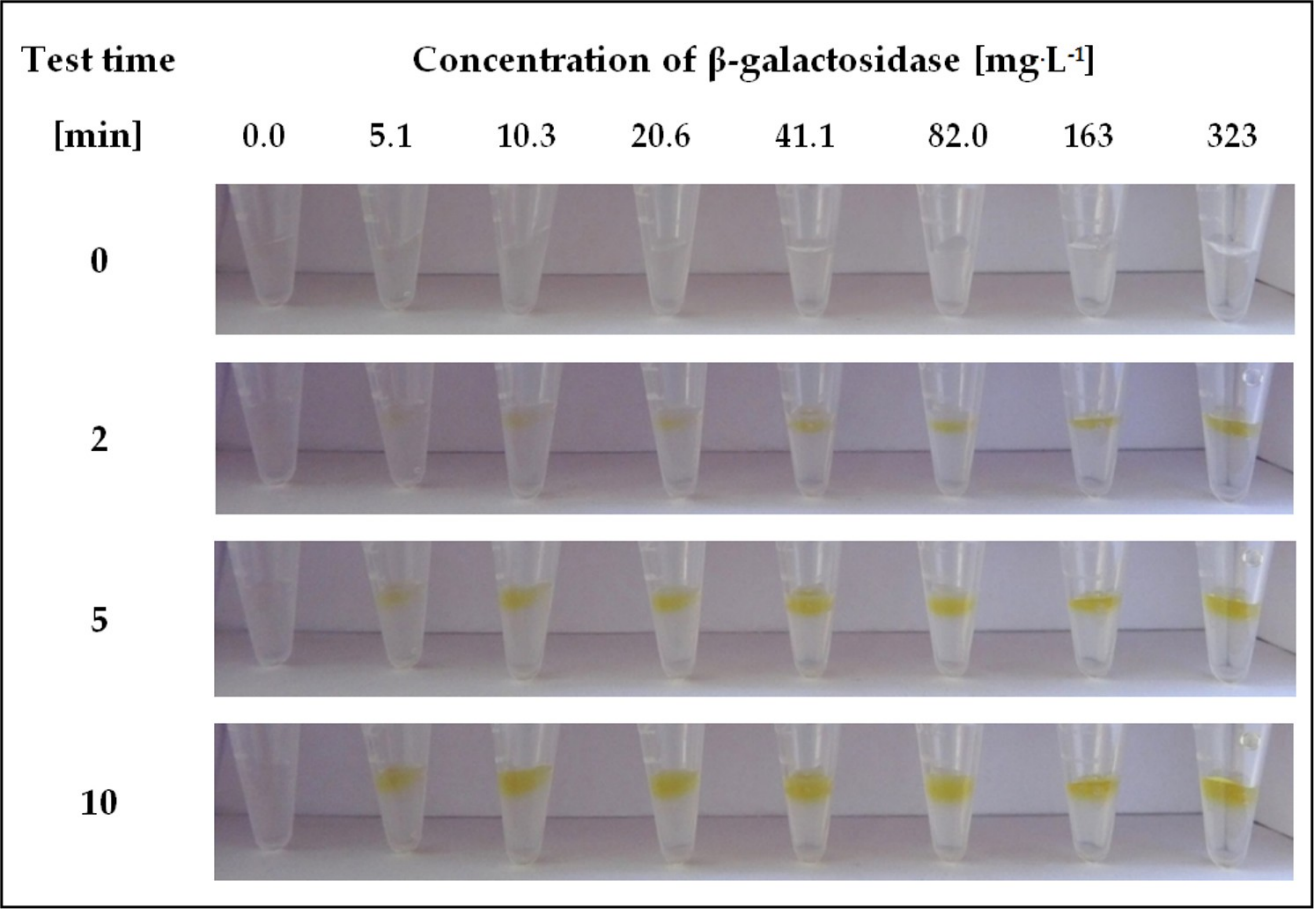
Visual response obtained after different times in the hydrogel-based test with immobilized ONPG performed for β-galactosidase in the concentration range of 5.1–323 mg^.^L^-1^.

**Fig 4:**
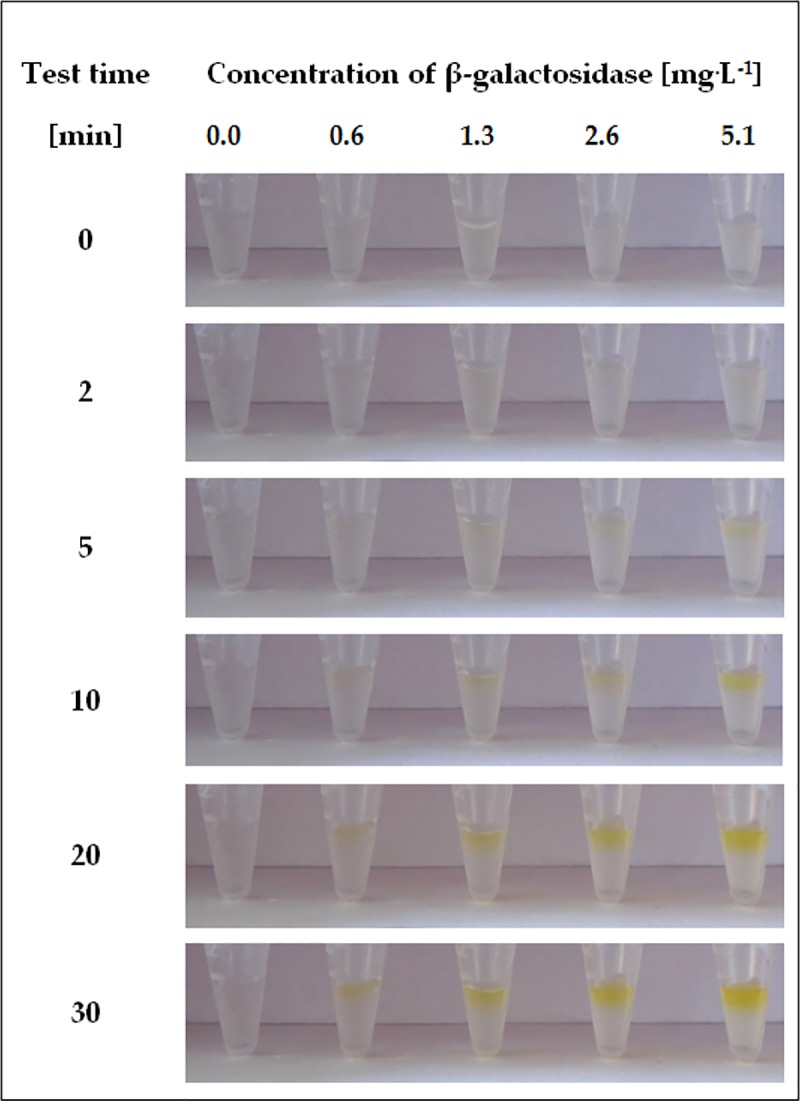
Visual response obtained after different times of the hydrogel-based test with immobilized ONPG, performed to test for β-galactosidase in the concentration range of 0.6–5.1 mg^.^L^-1^.

Detailed studies were performed to determine the sensitivity of the proposed diagnostic test. For this purpose, different concentrations of β-galactosidase were used in detection assays based on ONPG immobilized within the crosslinked structure of a hydrogel support. The results of experiments performed at a range of biocatalyst concentrations 0.6–323 mg^.^L^-1^ are presented in Figs 3 and 4.

The results showed that at higher concentrations of β-galactosidase (Fig 3), a clear change in gel colour could be observed only 5 minutes after application of the enzyme solution. In addition, after 10 minutes of the test, the resulting yellow colour was already very intense for all samples and did not change significantly thereafter. In turn, for the lower range of β-galactosidase concentrations, a noticeable effect of all examined solutions was visible after 20 min, and the intensive response appeared only after prolonging the test time to 30 min. The intensity of the yellow colour has also been observed to be directly proportional to increases in the concentration of the detected enzyme.

**Fig 5:**
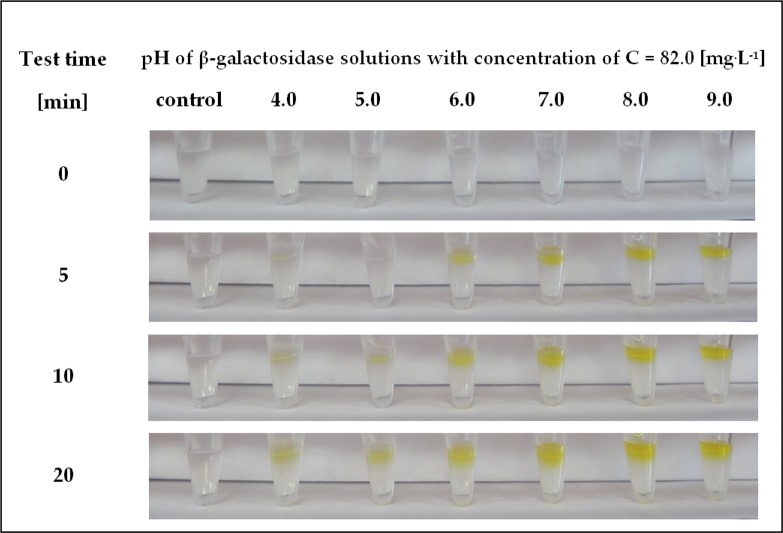
Visual response obtained after different times in the hydrogel-based test with immobilized ONPG performed at a constant concentration of β-galactosidase (82 mg^.^L^-1^) prepared in a pH range from 4.0 to 9.0.

β-Galactosidase from various sources may have different preferences for the acidity/basicity of the microenvironment [36–38]. Therefore, in the next step, the pH range of the biocatalyst preparations detectable by using the hydrogel-based assay containing immobilized ONPG was determined. For this purpose, enzyme solutions of pH 4.0 to 9.0 were prepared. Then, a small amount of each β-galactosidase solution was applied to the surface of the hydrogel matrix containing ONPG, and over time, a change in colour from transparent to yellow was observed. The results of the detection tests are shown in Fig 5.

The colour change became visible most rapidly, after only 5 min, for enzyme solutions with a pH of 6.0 to 9.0. In turn, 10 minutes after the start of the ONPG assay, the presence of β-galactosidase was detected in solutions throughout the pH range, and the yellow colour of all the samples only deepened over time.

Considering the economic aspect and the possibility of the commercial use of the test based on a hydrogel matrix with immobilized ONPG, a crucial feature is the possibility of the long-term storage of this product without loss of its specific properties.

**Fig 6:**
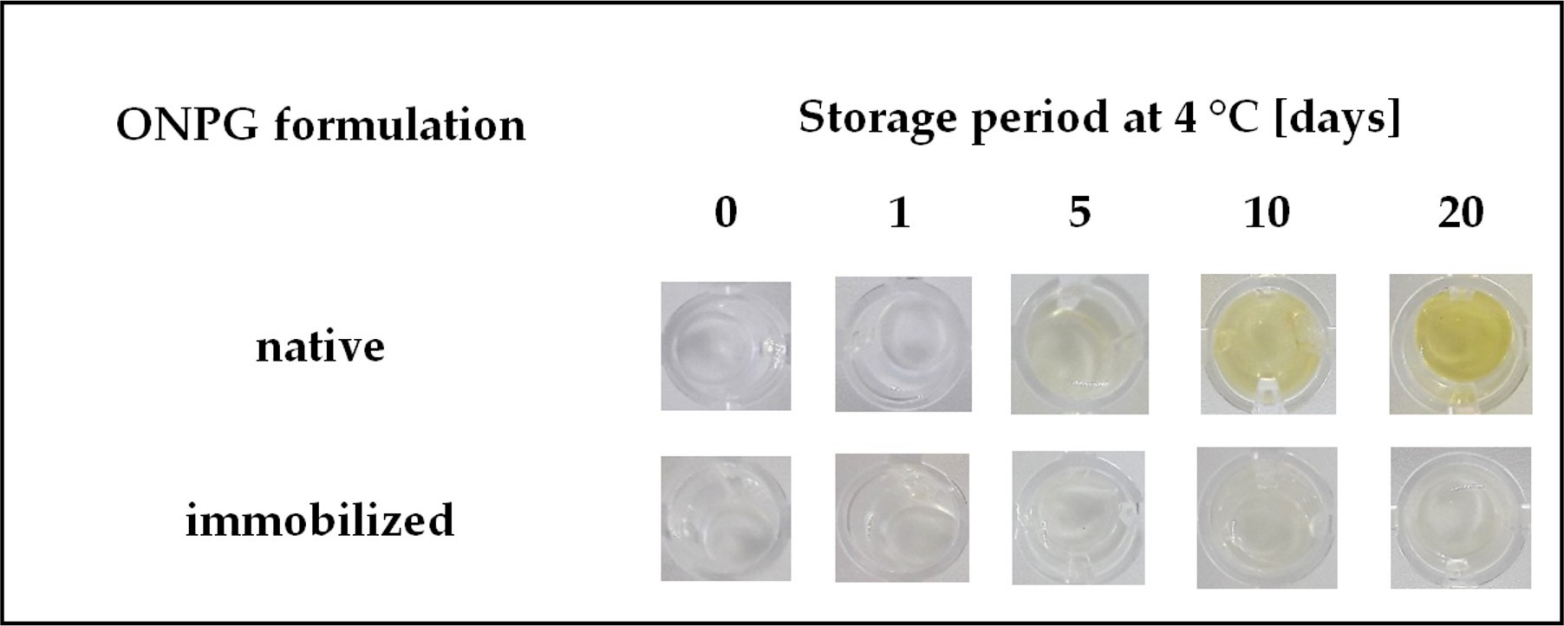
Storage stability of ortho-Nitrophenyl-β-D-galactopyranoside (ONPG) in native form and immobilized in the gelatine-based hydrogel matrix. Conditions: 4°C, 1–20 days.

The study began with examination of the storage stability of the ONPG solution and its immobilized form. The results presented in Fig 6 indicate that native ONPG is rather unstable just after 5 days storage at 4°C. Therefore, due to long-term storage stability, the application of ONPG entrapped inside cross-linked hydrogel matrix seems to be more preferable in this case.

Moreover, although the galactosidase detection test based on native form of ONPG results in a greater color intensity (S2 Appendix), the lack of background effects definitely makes that is more preferable to use ONPG immobilized in gelatine hydrogel.

**Fig 7:**
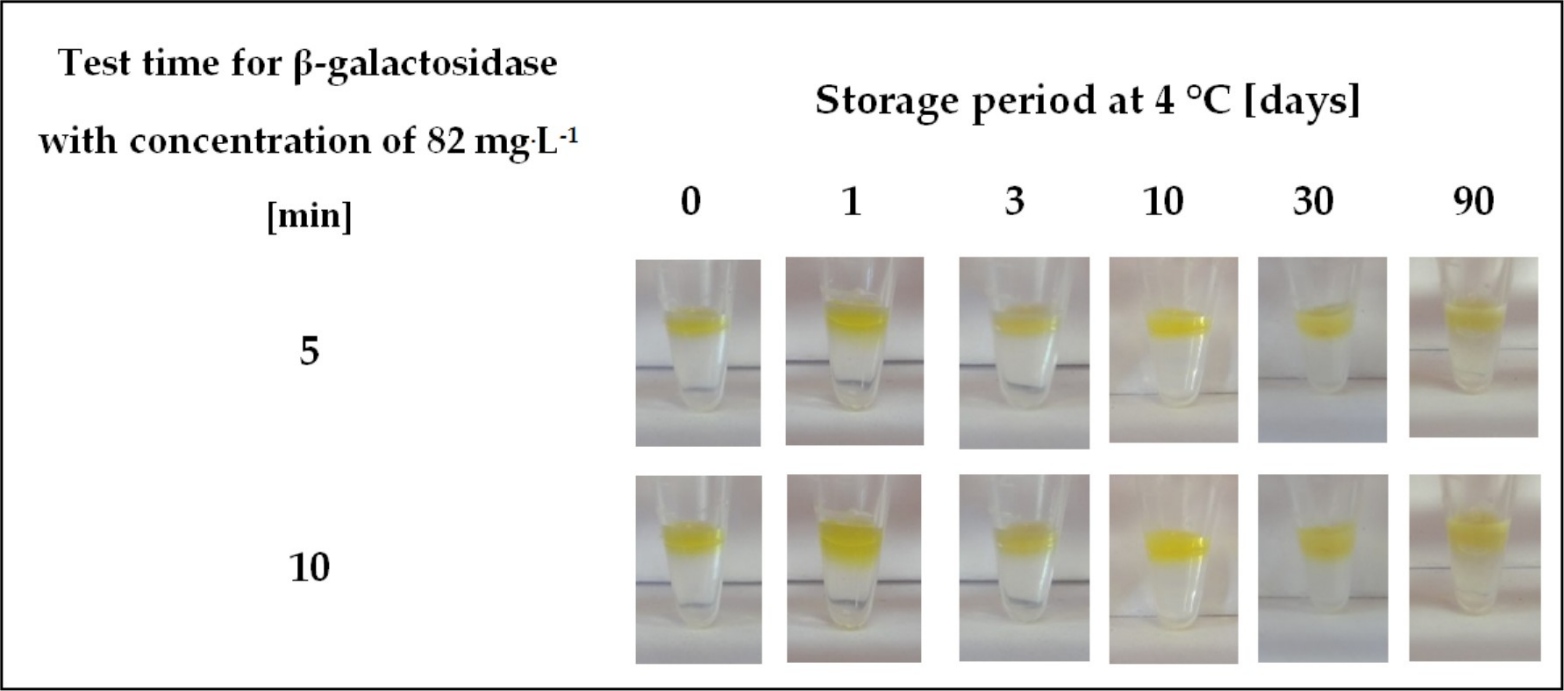
Storage stability of hydrogel-based test with immobilized ONPG. Conditions: 4°C, 1–90 days. Test properties were examined by carrying out the β-galactosidase detection assay for 5 and 10 minutes.

In the final phase of the research, the storage stability of the β-galactosidase detection test was determined. For this purpose, an assay for enzyme presence was performed by using ONPG-containing gelatine matrices that were first incubated in sealed Eppendorf tubes at 4°C from 1 to 90 days (Fig 7).

Results depicting the colourimetric response after a specified assay time showed that the hydrogel-based test with immobilized ONPG was stable over the entire range of storage periods.

To summarize the studies performed, the proposed colourimetric test for β-galactosidase detection possesses high application potential. The main advantages of gelatine hydrogel matrices containing ONPG include (i) the effective detection of the enzyme at concentrations greater than or equal to 0.6 mg^.^L^-1^, (ii) initial quantification of the enzyme according to the intensity of the obtained colour, (iii) applicability in a wide pH range (from 4.0 to 9.0), (iv) a relatively short response time (from 1 to a maximum of 30 minutes) and (v) the long-term storage stability at 4°C (90 days without loss of specific properties).

## Conclusions

A tangible result of the research performed in this study is a new, simple and rapid test for the detection of β-galactosidase–an effective biocatalyst for lactose hydrolysis. The proposed solution is based on the use of a specific substrate (ONPG) entrapped in a crosslinked hydrogel matrix. Among other factors, the range of β-galactosidase concentrations that could be effectively detected, the operational pH range, the time necessary to achieve a visible response and the preferred storage conditions of the test were determined in this study. In particular, the colourimetric test for β-galactosidase obtained as a result of these studies is distinguished by (i) the ease of making a gelatine hydrogel matrix, (ii) high selectivity and sensitivity, (iii) operation over a wide pH range, (iv) a relatively short response time and (v) the possibility of long-term storage without loss of its specific properties.

Based on these outcomes, gelatine hydrogel matrices with immobilized ONPG can be successfully used as: (i) a colourimetric diagnostic test selectively determining the presence and approximate concentration of β-galactosidase (e.g., for examination of the enzyme purity during successive steps of its production), (ii) for the differentiation of microorganisms (e.g., during the fermentation of lactose) and (iii) for marking microbial pathogens in water effluents of different origins.

The author assumes that in the future, the proposed solution may also contribute to provide an alternative diagnostic assay for lactose intolerance resulting from a lack of β-galactosidase (lactase) in the body. Because this ailment affects the majority of the adult worldwide population, the development of simple, rapid and selective assay for β-galactosidase determination would be of great interest for a modern medical diagnostics branch. In the examined assay based on ONPG immobilized in a crosslinked hydrogel matrix, a lack of colour response (no enzyme in the sample) would suggest further evaluation for lactose intolerance. Nevertheless, before the final confirmation of the effectiveness of this test, the proposed solution must be subjected to further research including the trials with body fluids and development of differentiation method between masking effect of ONPG indicator and a lack of color response due to lactose intolerance.

## Supporting information

S1 Appendix Kinetics parameters of the hydrolysis of *ortho*-nitrophenyl-β-D-galactopyranoside (ONPG) catalysed by β-galactosidase from *Kluyveromyces lactis* in native form. (DOCX)

S2 Appendix Storage stability of hydrogel-based test with native and immobilized ONPG. (DOCX)
