# Supplementary material for: Effective detection of biocatalysts with specified activity by using a hydrogel-based colourimetric assay – β-galactosidase case study
Source: PLoS One. 2018 Oct 11;13(10):e0205532. doi: 10.1371/journal.pone.0205532 (PMC6181394; doi:10.1371/journal.pone.0205532)
Supplement: S1 Appendix — (DOCX) [file pone.0205532.s001.docx]

S1 Appendix

Kinetics parameters of the hydrolysis of *ortho*-nitrophenyl-β-D-galactopyranoside (ONPG) catalysed by β-galactosidase from *Kluyveromyces lactis* in native form

Conditions of the kinetics studies: range of ONPG concentration 0.5 – 20 mM; β-galactosidase concentration 5.059 mg/L (determined using Lowry’s method); Thermostated batch reactor, 37°C)


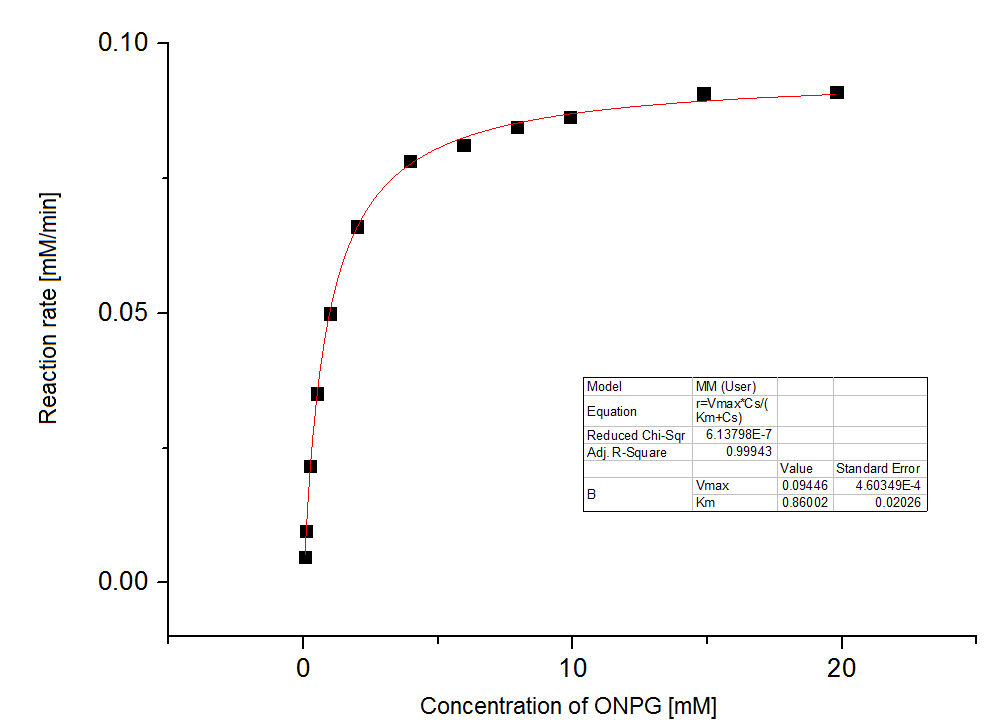


Fig. S1. Michaelis-Menten plot and the kinetics parameters (V_max_= 0.0945 ± 0.0005 mM/min; K_m_ = 0.8600 ± 0.0203 mM) determined using nonlinear regression (OriginPro).
