# Supplementary material for: Effective detection of biocatalysts with specified activity by using a hydrogel-based colourimetric assay – β-galactosidase case study
Source: PLoS One. 2018 Oct 11;13(10):e0205532. doi: 10.1371/journal.pone.0205532 (PMC6181394; doi:10.1371/journal.pone.0205532)
Supplement: S2 Appendix — (DOCX) [file pone.0205532.s002.docx]

S2 Appendix

Storage stability of hydrogel-based test with native and immobilized ONPG


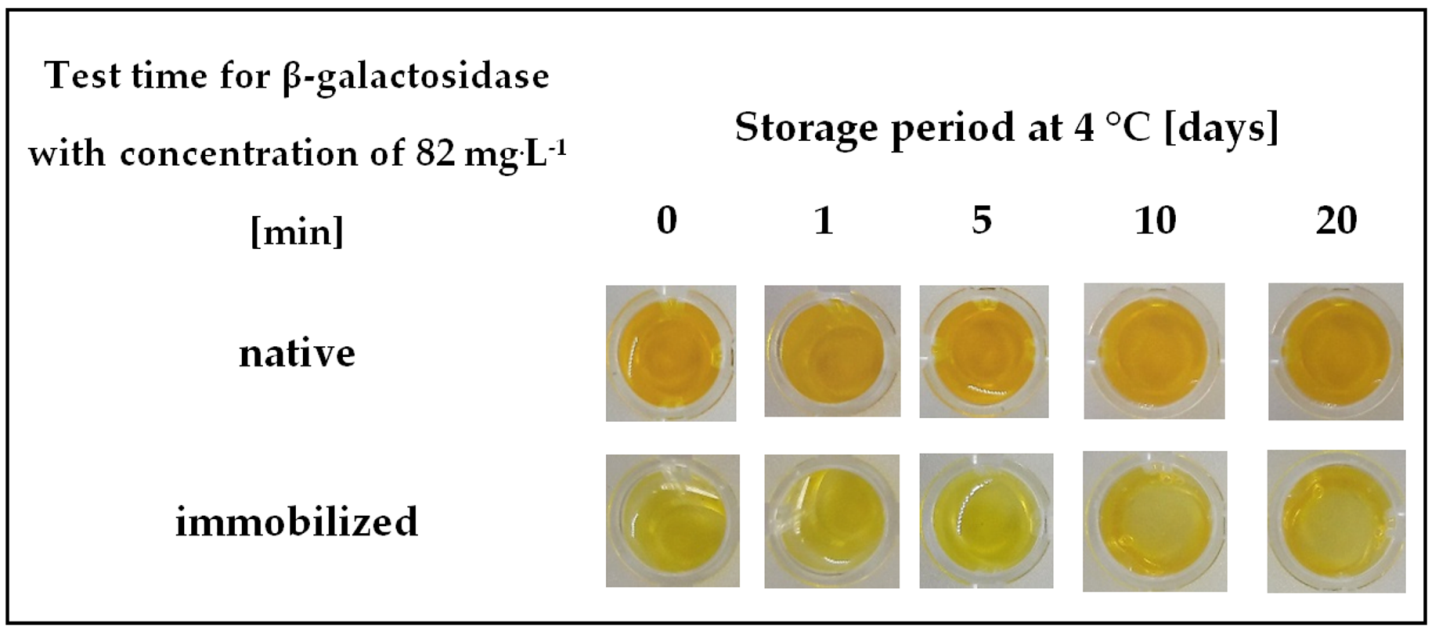


Fig. S2. Storage stability of hydrogel-based test with native and immobilized ONPG. Conditions: 4 °C, 1 – 20 days. Test properties were examined by carrying out the β-galactosidase detection assay for 10 minutes.
